# Adipokines levels in HIV infected patients: lipocalin-2 and fatty acid binding protein-4 as possible markers of HIV and antiretroviral therapy-related adipose tissue inflammation

**DOI:** 10.1186/s12879-017-2925-4

**Published:** 2018-01-05

**Authors:** Mario Luca Morieri, Viola Guardigni, Juana Maria Sanz, Edoardo Dalla Nora, Cecilia Soavi, Giovanni Zuliani, Laura Sighinolfi, Angelina Passaro

**Affiliations:** 1 Azienda Ospedaliero Universitaria di Ferrara, Via Aldo Moro 8, Cona, Ferrara, Italy; 2 Medical Science Department, University of Ferrara, Ferrara, Italy; 3 Unit of Infectious Diseases, Azienda Ospedaliero Universitaria di Ferrara, Cona, Ferrara, Emilia-Romagna Italy; 4 Department of Medical and Surgical Sciences, University of Bologna, Bologna, Italy

**Keywords:** Adipokines, HIV infections, Highly active antiretroviral therapy, Lipocalin-2, Adipose tissue, Inflammation, Fatty acid-binding proteins

## Abstract

**Background:**

Metabolic and cardiovascular diseases (CVD) represent a major problem in HIV infection. The aim of this study was to evaluate the relationship of HIV infection and antiretroviral therapy (ART) with circulating levels of two adipokines (Lipocalin-2 and Fatty Acid Binding Protein-4, FABP-4), known to be associated with adipose tissue dysfunction and cardiovascular disease in the general population.

**Methods:**

We enrolled 40 non-obese HIV-infected patients and 10 healthy controls of similar age and Body Mass Index (BMI). Body composition, metabolic syndrome, lipid profile, 10-years CVD risk score, and adipokines levels were compared between groups. ART-regimen status (naïve, non-nucleoside reverse transcriptase inhibitors – NNRTIs – and protease inhibitors – PIs) association with adipokines levels was tested with linear regression models.

**Results:**

HIV patients showed a worse metabolic profile than controls. Lipocalin-2 levels were higher in HIV-infected subjects (+53%; *p* = 0.007), with a significant trend (*p* = 0.003) for higher levels among subjects taking NNRTIs. Association of lipocalin-2 with fat-mass and BMI was modulated by ART regimens, being positive among subjects treated with NNRTIs and negative among those treated with PIs (“ART-regimens-by-BMI” interaction *p* = 0.0009). FABP-4 levels were correlated with age, fat mass, BMI, lipid profile and CVD risk (all *R* ≥ 0.32, *p* < 0.05), but not influenced by HIV-status (+20%; *p* = 0.12) or ART-regimen (*p* = 0.4).

**Conclusions:**

Our data confirm that HIV-infection is associated with adipose tissue inflammation, as measured by Lipocalin-2 levels, and ART does not attenuate this association. While FABP-4 is a marker of worse metabolic and CVD profile independently of HIV status or ART regimen, lipocalin-2 could represent a useful marker for HIV- and ART-related adipose tissue dysfunction.

**Electronic supplementary material:**

The online version of this article (10.1186/s12879-017-2925-4) contains supplementary material, which is available to authorized users.

## Background

In recent decades, the introduction and advances in Antiretroviral Therapy (ART) has dramatically decreased AIDS-related mortality among the HIV population, leading to an overall increase of longevity [1]. At the same time, HIV infected subjects are characterized by an early onset of age-related conditions demonstrating an accelerated aging process among these patients [2]. Consequently, care of subjects with HIV infection will increasingly require a multidisciplinary approach, with enforced attention focused on chronic co-morbidities, such as metabolic and cardiovascular diseases (CVD). HIV-associated metabolic disorders include abnormal adipose tissue distribution (HIV/ART-associated lipodystrophy syndrome - HALS), dyslipidemia, altered glucose metabolism and often overt metabolic syndrome [3–5].

Several specific pathways and factors are involved in the development of metabolic abnormalities in HIV-infected subjects. The use of antiretroviral drugs is associated with redistribution of adipose tissue and cardio-metabolic alterations, such as dyslipidemia [6, 7]. HIV infection itself may play a role through chronic inflammation and immune-activation mechanisms [8–10], and by altering adipose tissue function (e.g. in gene expression corresponding to mitochondrial function, adipocyte differentiation, and metabolism) [11]. The redistribution and hypo/hypertrophy of adipose tissue, accompanied by changes in its endocrine function (e.g. adipokines secretion), led to an accelerated atherogenesis [4, 12, 13]. Indeed HIV subjects showed a 2–3 fold increase of CVD compared to HIV-uninfected people, independently of other confounding factors [14, 15]. In conditions such as obesity or type 2 diabetes, dysfunctional adipose tissue becomes an important source of pro-inflammatory signals [16, 17]. Adipocytes and infiltrated macrophages produce adipokines that contribute to low-grade systemic inflammation leading to CVD [18]. Among adipokines, lipocalin-2 and fatty acid binding protein −4 (FABP-4), members of the superfamily of lipocalins, have been investigated as markers for adipose dysfunction associated with obesity and insulin resistance, and recently with coronary artery disease [16, 19–22]. Despite known adipose tissue dysfunction and increased CV risk in HIV-infected subjects [23], data on these adipokines in this population are scarce [24–28]. In particular, whether and how ART regimens may affect these cytokines is still an open question. Thus, the aim of this study was to evaluate the relationship of HIV infection and ART with circulating levels of lipocalin-2 and FABP-4, and to test the association of these adipokines with metabolic syndrome components (body fat distribution, lipid profile, insulin resistance) and with CVD risk among HIV-infected patients.

## Methods

In this mono-centric cross-sectional study, we included 40 HIV-infected patients, consecutively referred to our “Obesity, Diabetes, and Metabolic Syndrome outpatient service”, at S. Anna University Hospital (Ferrara, Italy), between January 2009 and March 2010. Exclusion criteria were the presence of active opportunistic infections and BMI > 30 kg/m^2^. Ten healthy non-obese HIV-uninfected healthcare practitioners from the same hospital were included as controls. All subjects underwent a complete medical interview and examination. They were weighed in light clothing; waist circumference was measured midway between the lower rib and the upper iliac crest, at the end of a normal expiration. Body composition was estimated by bioelectrical impedance analysis (Human IM plus II ®, DS Medica, Milan, Italy). Metabolic syndrome was defined by the presence of three or more updated National Cholesterol Education Program Adult-Treatment-Panel III (NCEP-ATPIII) criteria [29]: 1) waist circumference ≥ 102 cm in men or ≥88 cm in women; 2) triglycerides ≥150 mg/dL or treatment for hypertriglyceridemia; 3) HDL-cholesterol <40 mg/dL in men or <50 mg/mL in women or treatment for low HDL-C; 4) blood pressure ≥ 130/85 mmHg or treatment for hypertension; 5) fasting glucose ≥100 mg/dL or treatment for hyperglycemia. Subjects received a metabolic syndrome Score (MetS score), ranging from 0 (no features of metabolic syndrome) to 5 (all features). An HIV healthcare provider evaluated the presence of HALS according to existing definitions [3, 30]. Ten-year CVD risk was estimated with the Framingham Risk Equation based on age, total and HDL cholesterol levels, systolic blood pressure, the presence of diabetes, smoking status, and the presence of left ventricular hypertrophy on electrocardiography (set to zero in this analysis as data unavailable) [31].

### Biochemical analysis

Blood samples were collected after an overnight fasting. Plasma aliquots were stored at −80° until analytes of interest were assayed. Insulin resistance was calculated by HOmeostasis Model Assessment index (HOMA-IR): [(fasting insulin (U/L) × fasting glucose (mmol/L)) /22.5]. FABP-4 and lipocalin-2 were measured by ELISA Kit (BioVendor-Laboratory Medicine, Palackeho, Czech Republic), the detection limits of the assays were 0.1 ng/ml and 0.02 ng/ml, and the intra-assay coefficients were <6.0% and <8.9%, respectively. C-Reactive Protein was measured using a high sensitive immunotubidimeter test (Cobas Roche, Italy).

### Statistical analysis

Variables were expressed as mean ± S.D. or median and range interquartile. Variables with a non-normal distribution were log-transformed when appropriate before entering the statistical analysis. Differences between groups and variables of interest were analyzed with analysis of covariance (ANCOVA), with sex and age as covariates. When appropriate, the Fisher test was used to test the different prevalence of disease or condition (e.g. metabolic syndrome) between groups. The bivariate association between lipocalin-2, FABP4 and continuous variables of interest were tested by Spearman rank correlations. The modulation of treatment groups on the association between lipocalin-2 and BMI and fat mass, was tested with multivariate linear regression analysis, with the “treatment x BMI” interaction term, treatment and BMI as independent variables with age and sex as covariates. Tests were considered significant for a *p* value <0.05. According to power calculation, we expected (with a power of 80%) to detect a significant association (with alpha level = 0.05) for a 35% differences between HIV patients and controls in adipokines levels. All statistical tests were performed using SAS Institute Inc. (v 9.4). Graphs were edit using GraphPad Prism.

## Results

### Characteristics of the population

Table 1 reports the general characteristics of the population at baseline. Despite similar age, BMI and body composition, HIV patients showed worse metabolic profiles than controls, with higher glucose, non-HDL cholesterol, and hsCRP levels, associated with a higher prevalence of metabolic syndrome and higher CV risk (together with a non-significant trend for higher prevalence of hypertension). Among adipokines, subjects with HIV had on average a 52% higher levels of lipocalin-2 (*p* = 0.007) and a non-significant 19% higher levels of FABP-4 (*p* = 0.12).

**Table 1:** Characteristics of the population

Characteristics	HIVneg CTR (10)	HIV (40)	*P* value
Female (%)	5 (50%)	14 (35%)	0.4
Age (years)	45.3 ± 11.7	44.1 ± 8.2	0.8
Years since HIV diagnosis	n/a	3.5 (1.0–9.5)	–
HIV RNA > = 50 copies	n/a	13 (33%)	–
CD4 (cell/mm^3^)	n/a	608 (447–849)	–
BMI (kg/m2)	23.4 ± 2.9	24.4 ± 3.0	0.36
Waist circumference (cm)	85 ± 10	87 ± 9	0.99
Fat Mass (% of total mass)	21.8 ± 10.3	23.3 ± 8.6	0.25
Systolic Blood pressure (mmHg)	113 ± 8	119 ± 12	0.29
Diastolic Blood pressure (mmHg)	76 ± 5	80 ± 9	0.32
Hypertension	1 (10%)	16 (40%)	0.13
Fasting Blood Glucose (mg/dl)	87.3 ± 11.4	103.8 ± 19.8	0.02
Fasting Insulin (U/L)	6.6 (5.4–8.7)	6.0 (3.3–9.8)	0.70
HOMA-IR	1.5 (1.1–1.8)	1.5 (0.8–2.5)	0.83
Hyperglycemia	1 (10%)	21 (53%)	0.03
Tot-Cholesterol (mg/dl)	188 ± 35	217 ± 52	0.06
Triglycerides (mg/dl)	73 ± 20	176 ± 160	0.06
HDL-chol (mg/dl)	56 ± 14	48 ± 20	0.41
LDL-chol (mg/dl)	117 ± 28	133 ± 37	0.13
Non HDL-chol (mg/dl)	132 ± 31	168 ± 55	0.04
Metabolic Syndrome n (%)	0 (0%)	14 (35%)	0.03
HALS n (%)	–	12 (30%)	–
10 years CVD risk score (%)	1.5 (0.8–6.5)	8.0 (2.5–18.6)	0.01
hsCRP (mg/L)	1.1 (0.8–1.6)	2.0 (2.9–3.7)	0.001
Lipocalin-2 (ng/ml)	31.0 ± 7.9	47.3 ± 17.7	0.007
FABP4 (ng/ml)	13.1 ± 4.1	15.6 ± 7.6	0.12

Legend: *BMI* Body Mass Index, *HOMA-IR* homeostasis model assessment of insulin resistance, *HALS* HIV/ART Associated Lipodystrophy Syndrome, *CVD* Cardiovascular Diseases, *FABP4* Fatty Acid Binding Protein 4

According to treatment status, 10 HIV infected subjects were antiretroviral therapy-naïve, while 30 subjects were on PI-based (*n* = 13) or on NNRTI-based (*n* = 17) antiretroviral regimens (as reported in Additional file 1: Table S1). Most individuals taking PIs were female (69%), while among NNRTI and naïve group only three (18%) and two (20%) were women, respectively. Duration of HIV infection was similar between the three groups. Only three subjects on ART were not virologically suppressed (two on NNRTIs and one on PIs). The CD4 T-cells levels were similar among the groups (Additional file 1: Table S1). Among HIV-infected subjects, four were co-infected with HBV (specifically, one in the PIs group, two in the NNRTIs group and one in the naïve group), and none with HCV. Twelve subjects (30%) were diagnosed with HALS (specifically, eight with lipoatrophy, two with lipohypertrophy, two with mixed lipodystrophy).

### Metabolic syndrome, CVD risk and adipokines levels according to treatment status

**Fig. 1:**
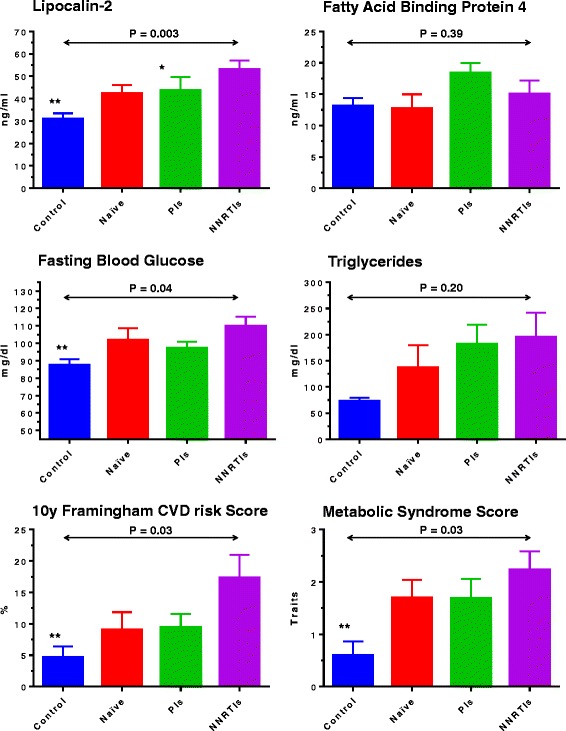
Circulating levels of adipokines, fasting plasma glucose and triglycerides, CV risk and metabolic syndrome components according to HIV-infected status and treatment regimen. Control (*n* = 10); Naïve (*n* = 10); PIs – subject with Protease Inhibitor based regimen (*n* = 13); NNRTIs – Non-Nucleoside Reverse Transcriptase Inhibitors based regimen (*n* = 17). Reported *P* value is the test for trend in the 4 groups (Post-hoc comparison vs NNRTIs * *P* < 0.05 and ** *P* < 0.01)

As shown in Fig. 1, the increase in lipocalin-2 levels among HIV subjects was not attenuated by HIV treatment, on the contrary, there was a significant trend (*p* = 0.003) for higher levels among subjects taking NNRTIs. The same group was also characterized by higher glucose levels (P for trend =0.04) and higher metabolic syndrome components (P for trend =0.03). According to the 10-year Framingham CV risk score, this group was also at higher CV risk (P for trend =0.03). Conversely, lipid profiles and blood pressure were similar between groups (Additional file 1: Table S1). Levels of hsCRP, that were overall higher among HIV infected subjects, were similar across treatment groups. Also BMI levels were similar between subjects on PIs- NNRTIs- or naïve- treatment (BMI ± S.D. were 24.6 ± 3.7, 24.5 ± 3.1, and 24.1 ± 1.9 (Kg/m^2^) respectively; P for differences = 0.6). No relevant differences in FABP-4 levels were observed between groups.

### Determinants of adipokines levels in HIV patients and influence of treatment regimens

Table 2 shows that among subjects with HIV higher levels of lipocalin-2 were not associated with body composition, CVD risk, glycemic or lipid profile (all coefficient of correlation <0.14). In contrast, FABP4 levels showed a strong positive correlation with age, fat mass and BMI (all *R* ≥ 0.48, *p* < 0.005), and a moderate positive correlation with levels of triglycerides, non-HDL-cholesterol, MetS Score and CVD risk (all *R* ≥ 0.32, *p* < 0.05). Neither lipocalin-2 nor FABP4 levels were significantly correlated with CD4 T cells count, HIV infection duration, HIV RNA or with hsCRP.

**Table 2:** Spearman rank correlation of Lipocalin-2 (LCN2) and Fatty Acid Binding Protein 4 (FABP4) levels with body composition, CVD risk, glycemic and lipid profile among HIV-infected patients

	FABP4	Age	BMI	Fat Mass	Glucose	HOMA IR	HDL-c	TG	Non HDL-c	LDL-c	MetS Score	CVD risk	CD4 count	HIV duration	hsCRP	HIV Rna
LCN2	0.10	−0.04	−0.09	0.01	0.02	−0.01	0.04	−0.10	0.06	0.13	0.03	0.12	0.13	0.06	−0.30	−0.04
FABP4		0.48^	0.57ª	0.73ª	0.18	0.14	−0.05	0.32°	0.42°	0.31	0.35°	0.33°	−0.01	0.01	0.03	−0.24
Age	–		0.26	0.46^	0.08	−0.12	0.13	0.00	0.36°	0.50^	0.14	0.56^	−0.21	0.15	0.18	−0.20
BMI	–	–		0.76ª	0.15	−0.02	−0.09	0.39°	0.50^	0.34°	0.42°	0.41°	0.06	0.15	−0.03	−0.17
Fat Mass	–	–	–		0.05	−0.02	0.10	0.30	0.50^	0.42°	0.36°	0.39°	0.14	0.21	0.00	−0.18
Glycemia	–	–	–	–		0.51^	−0.29	0.31	0.28	−0.02	0.64ª	0.55^	−0.02	0.18	−0.07	0.08
HOMA-IR	–	–	–	–	–		−0.55^	0.57ª	0.28	−0.04	0.52^	0.31	0.06	0.00	−0.22	0.24
HDL-c	–	–	–	–	–	–		−0.60ª	−0.36°	−0.02	−0.52^	−0.39°	0.14	0.21	−0.08	−0.03
TG	–	–	–	–	–	–	–		0.64ª	0.10	0.67ª	0.36°	0.11	0.00	−0.15	−0.16
Non HDL-c	–	–	–	–	–	–	–	–		0.77ª	0.47^	0.52^	0.11	0.26	−0.17	−0.10
LDL-c	–	–	–	–	–	–	–	–	–		0.03	0.36°	0.13	0.32°	−0.17	−0.06
MetS Score	–	–	–	–	–	–	–	–	–	–		0.76ª	−0.08	0.09	−0.06	−0.07
CVD risk	–	–	–	–	–	–	–	–	–	–	–		−0.09	0.26	0.01	−0.08
CD4 cell	–	–	–	–	–	–	–	–	–	–	–	–		0.50^	−0.42°	−0.14
HIV Duration	–	–	–	–	–	–	–	–	–	–	–	–	–		−0.07	−0.19
hsCRP																−0.22

Legend: *BMI* Body Mass Index, *HOMA-IR* homeostasis model assessment of insulin resistance, *CVD* Cardiovascular Diseases, *CRP* C-reactive Protein

° *P* < .05; ^ *P* < .005; ª *P* < 0.0001

**Fig. 2:**
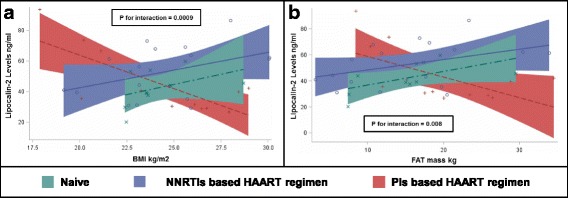
Linear association between lipocalin-2 and body composition (BMI in Panel **a** and FAT mass in Panel **b**) according to ART regimen among HIV infected patients. Green dashed line = subjects Naïve to treatment (*n* = 10); Red dashed line = subjects treated with Protease Inhibitor (PIs) based ART regimen (*n* = 13); Blue continuous line = subjects treated with Non-Nucleoside Reverse Transcriptase Inhibitors (NNRTIs) based ART regimen (*n* = 17); Legend: BMI = Body Mass Index

**Fig. 3:**
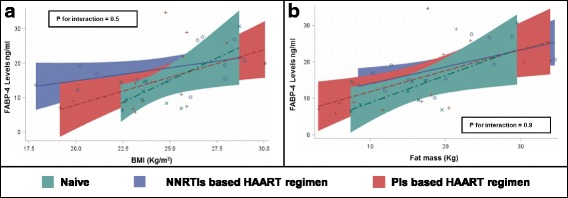
Linear association between Fatty Acid Binding Protein 4 (FABP4) and body composition (BMI in Panel **a** and FAT mass in Panel **b**) according to ART regimen among HIV infected patients. Green dashed line = subjects Naïve to treatment (*n* = 10); Red dashed line = subjects treated with Protease Inhibitor (PIs) based ART regimen (*n* = 13); Blue continuous line = subjects treated with Non-Nucleoside Reverse Transcriptase Inhibitors (NNRTIs) based ART regimen (*n* = 17); Legend: BMI = Body Mass Index

Since we found a trend for different lipocalin-2 levels according to patients’ treatment status, we evaluated whether ART-regimens could influence the relationship between lipocalin-2 and measures of adipose tissue accumulation. Interestingly, as shown in Fig. 2-A, we found that the association between BMI and lipocalin-2 levels was modulated by treatment status (P for interaction = 0.0009, age and sex-adjusted). Indeed among naïve subjects and those treated with NNRTIs, lipocalin-2 levels increased with increasing BMI and fat mass (Fig. 2-B), while among subjects treated with PIs, there was the opposite trend (with lower levels of lipocalin-2 in those subjects having higher BMI). Further exploratory analyses showed that these influences were confirmed when the population was stratified by median duration of HIV infection (i.e. 3.5 years, P for interactions ≤0.05 in both groups). In contrast, FABP-4 association with adipose tissue accumulation was similar across treatment groups (Fig. 3 A-B).

## Discussion

With the introduction and the success of ART, HIV infection has become a chronic disease [1]. Therefore, there are growing concerns about long-term metabolic toxic effects derived from exposure to antiretroviral drugs and on consequences of HIV-related chronic inflammation that can lead to complications such as CVD. Adipose tissue is involved in the production of cytokines with pro- and anti-inflammatory properties (i.e. adipokines), but also represents a target for the antiretroviral drugs in the context of ART-related side effects (e.g. lipodystrophy). For these reasons, changes in adipose tissue function are of great interest for the management and care of subjects with HIV infection. In this study, we analyzed the association between HIV infection and ART with the levels of two adipokines, lipocalin-2 and FABP-4. We found that HIV status and ART regimen had an important role in the modulation of lipocalin-2 levels, while the influence on circulating levels of FABP-4 was very modest to none. Furthermore, to our knowledge, this is the first study in which an association between lipocalin-2 levels, as an expression of adipose tissue inflammation, and different ART-regimens has been described.

As expected [13–15], we found that HIV subjects, were characterized by higher glucose levels, worse lipid profile, higher prevalence of metabolic syndrome and consequently higher CVD risk, when compared to uninfected individuals with similar BMI and age. HIV-associated metabolic disturbances seem to have a major role in the possible higher CV risk in HIV subjects, and this role seems to be mediated, at least in part, by circulating adipokines. In the general population levels of Lipocalin-2, have been reported to be increased in conditions such as obesity, metabolic syndrome and cardiovascular disease [19, 21, 32–34]. Conversely, there is a lack of data on the relationship between this adipokine and metabolic parameters among subjects infected with HIV.

Interestingly we found that lipocalin-2 levels were significantly higher in HIV subjects compared to controls, although we did not find any significant association between this adipokine and metabolic syndrome components. On the other hand, FABP-4 levels were influenced neither by HIV infection nor by ART-regimens but were associated with body composition, lipid profile, MetS score and estimated CVD risk (as previously reported both in subjects with and without HIV infection) [22, 24, 26].

Furthermore, lipocalin-2 levels were different among HIV-treatment groups, being higher in the subjects treated with NNRTIs. Surprisingly, even though this group was characterized by a higher number of metabolic syndrome components and by a higher estimated CV risk, lipocalin-2 itself was not associated with these traits (in contrast to the positive correlation that has been previously reported in the non-HIV-infected population [19]). As a possible clarification of these counterintuitive findings, we found that the degree of the association between lipocalin-2 levels and measures of accumulation and distribution of adipose tissue varied according to treatment regimens. Indeed, the expected positive correlation between fat mass and lipocalin-2 levels was found only in the NNRTIs-treated group but not in the PIs treated group, which showed a negative correlation.

Lipocalin-2 is well known to be up-regulated in the inflammatory response (e.g. by direct NF-kb related transcription activation) [35], with higher levels being associated with inflammation of adipose tissue [19]. Despite this, it is not yet clear if the increase in lipocalin-2 in these conditions mediates pro-[20, 34] or anti-inflammatory effects [36–38]. Given the possible compensatory role of this cytokine, and given the cross-sectional design of our study, caution must be exercised in the interpretation of these findings, and different hypothesis should be considered.

In the first scenario, the increased levels of lipocalin-2 among NNRTIs-treated subjects (positively correlated with adipose tissue accumulation), could be seen as a sign of higher or different adipose tissue inflammation. Indeed, among NNRTI-treated HIV subjects, 16 out of 17 were treated with Efavirenz (EFV), a drug with recognized pro-inflammatory activity [39]. As reported by Domingo et al. [40], the analysis of subcutaneous adipose tissue of HIV-infected patients has shown increased transcription of pro-inflammatory genes (e.g. MPC-1, TNF-alpha, IL-18) in EFV-treated group compared to Lopinavir-treated group. Remarkably, body composition changes were similar in the two groups [40]. Other in vitro studies on adipocytes and clinical studies reported higher levels of secretion and circulation of inflammatory cytokines induced by NNRTIs regimen compared to PIs regimen [9, 41]. Also, Landro et al. found that lipocalin-2 levels increase over time after initiating ART [42], but they did not analyze the effect of different ART-regimens. Similarly to our study, Allavena et al. found a significant difference in plasma lipocalin-2 levels according to different ART-regimens (although they tested abacavir/lamivudine Vs tenofovir/emtricitabine, within subjects treated with the same NNRTI).

In a different scenario, given the well-known worse cardiovascular safety profile of PIs [43], the inverse association of lipocalin-2 with adipose tissue accumulation among PIs-treated subjects, might be consistent with the compensatory-anti-inflammatory effect of this adipokine. In other words, a lesser increase of lipocalin-2 with adipose tissue accumulation and dysfunction might be a sign of a different, and maybe more atherogenic, inflammatory response of adipose tissue among subjects treated with PIs.

All these findings, although preliminary, pave the way for understanding possible ART-regimen-specific differences in the inflammation response of the adipose tissue, involving the expression and secretion of lipocalin-2 but not of FABP-4.

Despite this promising hypothesis, alternative explanations and limits must be considered. In our study, the NNRTI-treated group was characterized by a higher prevalence of metabolic syndrome, higher CV risk and higher glucose levels. However, given the cross-sectional design, a confounding by indication might have influenced these associations. Indeed, in clinical practice, this could be explained by the fact that NNRTIs, rather than PIs, are commonly considered safe drugs with respect to their effects on adipose tissue and are more often prescribed to patients with some degree of metabolic disturbances and higher CVD risk. Thus, the differences between the inflammatory profiles of adipose tissue across treatment groups could be linked to unknown underlying factors more strongly than to the treatment itself. In addition, the sample size could have limited our ability to find a statistical relevance for the 20% higher levels of FABP-4 in HIV-infected patients compared to controls. Finally, no data on HIV integrase inhibitors (the newest antiretroviral drug class) are available, since at the time of data collection none of the included subjects was taking these drugs as a part of the ART regimen. Although NNRTI and PI-based regimens are currently still largely used, evaluation of the association of these adipokines with more recent anti-HIV drugs will be important.

## Conclusions

In conclusion, we found that HIV itself was associated with higher adipose tissue inflammation, and such response, as measured by circulating levels of lipocalin-2, was not reversed by ART. Conversely, our data highlight the possible role of lipocalin-2 as a marker of the different inflammatory effect of NNRTIs and PIs on adipose tissue. Further studies are required to investigate the possible causality of this relationship and to evaluate the role of this adipokine as a marker of metabolic dysfunction and CVD risk in HIV infected subjects.

## Additional file

Additional file 1: Table S1. General characteristics of the population according to treatment status. (DOCX 15 kb)

## Abbreviations

AIDS: Acquired Immune Deficiency Syndrome
ART: Antiretroviral Therapy
BMI: Body Mass Index
CVD: Cardiovascular Disease
EFV: Efavirenz
FABP-4: Fatty Acid Binding Protein-4
HALS: HIV/ART-associated lipodystrophy syndrome
HBV: Hepatitis B Virus
HCV: Hepatitis C Virus
HDL: High-Density Lipoprotein
HIV: Human Immunodeficiency Virus
HOMA-IR: HOmeostasis Model Assessment - Insulin Resistance index
hsCRP: high sensitivity C-reactive protein
LCN-2: Lipocalin-2
MetS: Metabolic Syndrome
NCEP-ATPIII: National Cholesterol Education Program Adult-Treatment-Panel III
NNRTIs: Non-Nucleoside Reverse Transcriptase Inhibitors
PIs: Protease Inhibitors.
