# Supplementary material for: Adipokines levels in HIV infected patients: lipocalin-2 and fatty acid binding protein-4 as possible markers of HIV and antiretroviral therapy-related adipose tissue inflammation
Source: BMC Infect Dis. 2018 Jan 5;18:10. doi: 10.1186/s12879-017-2925-4 (PMC5756414; doi:10.1186/s12879-017-2925-4)
Supplement: Additional file 1: Table S1. — General characteristics of the population according to treatment status. (DOCX 15 kb) [file 12879_2017_2925_MOESM1_ESM.docx]

**Additional file 1**

**Table S1:** general characteristics of population at baseline according to treatment status

| Characteristics | CTR (10) | Naïve (10) | PI (13) | NNRTI (17) | P value |
| --- | --- | --- | --- | --- | --- |
| Female (%) | 5 (50%) | 2 (20%) | 9 (69%) † | 3 (18%) | 0.03 |
| Age Years | 45.3 ± 11.7 | 41.2 ± 8.0 | 45.3 ± 6.6 | 44.8 ± 9.4 | 0.7 |
| Years Since HIV Diagnosis | - | 2.0 (1.0-6.0) | 4.0 (2.0-8.0) | 5.0 (1.0-11.0) | 0.8* |
| HIV RNA >= 50 Copies | - | 10 (100%) | 2 (15%) | 1 (6%) | <0.0001* |
| CD4 (cell count) | - | 539 (454-713) | 715 (495-882) | 603 (418-777) | 0.6* |
| BMI (kg/m^2^) | 23.4 ± 2.9 | 24.1 ± 1.9 | 24.6 ± 3.7 | 24.5 ± 3.1 | 0.69 |
| Waist Circumference (cm) | 85 ± 10 | 86 ± 6 | 85 ± 12 | 89 ± 8 | 0.86 |
| Fat Mass (%) | 21.8 ± 10.3 | 21.7 ± 8.4 | 27.7 ± 6.9 | 20.9 ± 9.0 | 0.49 |
| Systolic BP (mmHg) | 113 ± 8 | 120 ± 8 | 112 ± 6 | 123 ± 14 | 0.11 |
| Diastolic BP (mmHg) | 76 ± 5 | 80 ± 9 | 77 ± 8 | 83 ± 9 | 0.30 |
| Hypertension n (%) | 1 (10%) | 5 (50%) | 2 (15%) | 9 (53%) |  |
| Blood Glucose (mg/dl) | 87.3 ± 11.4 ‡ | 101.9 ± 21.4 | 97.2 ± 13.7 | 110.0 ± 21.9 | 0.04 |
| Fasting Insulin (UI/l) | 6.6 (5.4-8.7) | 5.3 (4.1-13.4) | 6.3 (3.1-8.4) | 6.1 (3.2-8.7) | 0.96 |
| HOMA-IR | 1.5 (1.1-1.8) | 1.3 (1.0-3.9) | 1.5 (0.7-2.1) | 1.9 (0.8-2.5) | 0.95 |
| Hyperglycemia (MetS criteria) | 1 (10%) | 4 (40%) | 5 (38%) | 12 (71%) |  |
| Tot-Cholesterol mg/dl | 188 ± 35 | 214 ± 66 | 218 ± 45 | 217 ± 51 | 0.28 |
| Triglycerides mg/dl | 73 ± 20 | 137 ± 137 | 182 ± 134 | 195 ± 193 | 0.20 |
| HDL-Chol mg/dl | 56 ± 14 | 52 ± 20 | 47 ± 18 | 47 ± 22 | 0.22 |
| LDL-Chol mg/dl | 117 ± 28 | 135 ± 49 | 135 ± 33 | 131 ± 35 | 0.43 |
| Non HDL-Chol mg/dl | 132 ± 31 | 162 ± 68 | 171 ± 49 | 170 ± 53 | 0.23 |
| Metabolic Syndrome components | 0.6 ± 0.84 | 1.7 ± 1.3 | 1.7 ± 1.1 | 2.2 ± 1.4 | 0.03 |
| HALS n (%) | - | 2 (20%) | 5 (38 %) | 5 (29%) | 0.5* |
| 10 Years CVD Risk Score (%) | 1.5 (0.8-6.5) ‡ | 7.0 (2.5-15.4) | 8.2 (3.3-11.7) | 14.3 (3.5-26.9) | 0.03 |
| Lipocalin-2 (ng/ml) | 31.0 ± 7.9 ‡ | 42.4 ± 11.8 | 43.7 ± 21.1 † | 52.9 ± 17.1 | 0.003 |
| FABP-4 (ng/ml) | 13.1 ± 4.1 | 12.8 ± 7.1 | 18.4 ± 5.8 | 15.1 ± 8.8 | 0.39 |

Legend: BMI, Body Mass Index ; HOMA-IR, homeostasis model assessment of insulin resistance; HALS, HIV/ART Associated Lipodystrophy Syndrome; CVD, Cardiovascular Diseases; FABP4, Fatty Acid Binding Protein 4. * P value computed only across HIV-infected groups; Post-hoc comparison with NNRTIs † P<0.05 ; ‡ P<0.01.
